# Aneurism of the Aorta

**Published:** 1845-01

**Authors:** 


*Aneurism of the Aorta.*—In a very interesting paper on this subject, Dr. Law draws the following inferences:—

"When aneurism arises from the posterior part of the aorta, we generally want the evidence of a palpable tumor to indicate the disease. When the tumor is resisted in its development by unyielding structures, (as in the case when it arises from the posterior part of the aorta) it produces in these structures changes giving rise to peculiar symptoms, especially to a peculiar character of pain, which, if not exclusively confined to this disease, exists so much more frequently in it than in any other, as to be enough at all times to awaken a suspicion of aneurism. However obscure all other symptoms of aneurism of the aorta, apart from the existence of a palpable tumor, may be, still it rarely happens that there are not some, which, added to the existence of the particular pain, may not suffice to make up what this latter may want of an exclusive patho-gnomonic sign of the disease. If this pain be connected with the lower dorsal and lumbar vertebræ, and depend upon abdominal aneurism, there will be, according to our constant experience, a bruit de soufflet in the course of the artery. If the pain be connected with the upper or thoracic dorsal vertebræ, and be owing to aneurism, it seldom occurs that there is not some difficulty in deglutition, or some obstruction in the respiratory apparatus, either affecting the trachea, and so weakening the respiration in both lungs, or exercised upon either one bronchus or upon one lung, and so producing a difference in the relative form of the respiration in the two lungs. In the absence of the bruit de soufflet (which we have almost always found absent in thoracic aneurism, except where the valves of the aorta were involved in the disease), some one of these symptoms will generally be present to confirm the value of the pains.

The character of the pain consists in a constant, aching, boring sensation, and a sharp, lancinating pain. To relieve the agonizing pain of aneurism, there is scarcely a limit to the amount to which we may exhibit opium, without producing narcotism. In the treatment of aneurism, low diet should be avoided, as lessening the prospect of a radical cure of the disease, and as increasing a nervous irritability—the constant accompaniment of it. The interval between the fatal termination and the bursting of an aneurism is various, and is much influenced by the importance of the organs which the hæmorrhage may affect. If it burst into the pericardium, and compress the heart, such interval will, of course, be shorter than if it compress a less vital organ. If there have been an adhesion between the laminæ of the pericardium the effusion will be more gradual, and therefore the interval will be longer than if no such adhesion existed, as we have proved by experience. The suddenness of the fatal termination would seem to be in proportion to the extent and suddenness of the hæmorrhage, and the importance of the organs, whose function may be mechanically interfered with by the effused blood.—*Dublin Jour. of Med. Science.—From Braithwaite's Retrospect.*

